# Mangiferin Improved Palmitate-Induced-Insulin Resistance by Promoting Free Fatty Acid Metabolism in HepG2 and C2C12 Cells via PPAR*α*: Mangiferin Improved Insulin Resistance

**DOI:** 10.1155/2019/2052675

**Published:** 2019-01-27

**Authors:** Qiao Zhang, Xiangju Kong, Hang Yuan, Hongjun Guan, Ying Li, Yucun Niu

**Affiliations:** ^1^Department of Nutrition and Food Hygiene, Public Health College, Harbin Medical University, Harbin 150086, China; ^2^Department of Gynaecology, First Affiliated Hospital of Harbin Medical University, Harbin 150000, China; ^3^Public Health College, Mudanjiang Medical University, Mudanjiang 157011, China

## Abstract

Elevated free fatty acid (FFA) is a key risk factor for insulin resistance (IR). Our previous studies found that mangiferin could decrease serum FFA levels in obese rats induced by a high-fat diet. Our research was to determine the effects and mechanism of mangiferin on improving IR by regulating FFA metabolism in HepG2 and C2C12 cells. The model was used to quantify PA-induced lipid accumulation in the two cell lines treated with various concentrations of mangiferin simultaneously for 24 h. We found that mangiferin significantly increased insulin-stimulated glucose uptake, via phosphorylation of protein kinase B (P-AKT), glucose transporter 2 (GLUT2), and glucose transporter 4 (GLUT4) protein expressions, and markedly decreased glucose content, respectively, in HepG2 and C2C12 cells induced by PA. Mangiferin significantly increased FFA uptake and decreased intracellular FFA and triglyceride (TG) accumulations. The activity of the peroxisome proliferator-activated receptor *α* (PPAR*α*) protein and its downstream proteins involved in fatty acid translocase (CD36) and carnitine palmitoyltransferase 1 (CPT1) and the fatty acid *β*-oxidation rate corresponding to FFA metabolism were also markedly increased by mangiferin in HepG2 and C2C12 cells. Furthermore, the effects were reversed by siRNA-mediated knockdown of PPAR*α*. Mangiferin ameliorated IR by increasing the consumption of glucose and promoting the FFA oxidation via the PPAR*α* pathway in HepG2 and C2C12 cells.

## 1. Introduction

Insulin resistance (IR) is a physiological condition in which cells fail to respond to the normal actions of the hormone insulin [1]. The body produces insulin, but the cells in the body become resistant to it and are unable to use it as effectively, leading to high blood glucose [2]. Elevated plasma-free fatty acid (FFA) is a risk factor for IR and type 2 diabetes mellitus (T2DM) [3]. An excess of FFA in the blood causes increased accumulation of lipid metabolites in the liver and skeletal muscle and can further worsen IR, which is the core defect in T2DM. Furthermore, FFA and their metabolites can also interfere with insulin signaling and inhibit insulin-stimulated glucose uptake and glycogen synthesis [4]. Therefore, lowering the blood FFA levels and reducing the lipid metabolite accumulations of peripheral tissues have been considered an effective strategy to improve IR and diabetes.

Important sites of FFA removal from the blood are the liver at rest and the skeletal muscle during activity [5]. In glucose and lipid metabolic disorders, lipid droplet accumulations in the liver and skeletal muscle can raise the FFA levels in the blood, which increases the risk of hypertension, atherosclerosis, and cardiovascular disease, including IR and T2DM [6]. In addition, skeletal muscle is the primary site for insulin-stimulated glucose disposal and is susceptible to impaired insulin action by elevated fatty acid availability in the human body [7], accounting for 80%–90% of all the glucose taken up from the blood. Therefore, it is a proposed strategy for mitigating IR to promote plasma FFA transfer to the liver and the skeletal muscle and to promote oxidation of FFA transferred rather than accumulated in these tissues.

Mangiferin is a natural plant chemical and exists in many kinds of plants and Chinese herbal medicines such as *Anemarrhena asphodeloides*, *Mangifera indica*, and *Mangifera persiciformis* [8, 9]. Mangiferin has plenty of beneficial biological activities, such as anti-inflammatory, antioxidant, hypolipemic, and antihyperglycemic effects [9–11]. In addition, our studies found that mangiferin had the effect of decreasing serum triglycerides (TG) and FFA levels in hyperlipidemic hamsters and rats by inhibiting lipogenesis and promoting fatty acid oxidation [12]. Furthermore, some studies have shown that mangiferin may improve IR both *in vivo* and *in vitro* [13]. However, the mechanism by which mangiferin mitigated IR caused by FFA metabolism remains unclear. The aim of our study was to explore the effects and mechanism of mangiferin on IR in both HepG2 and C2C12 cells.

## 2. Materials and Methods

### 2.1. Reagents

Dulbecco's Modified Eagle's Medium (DMEM) was purchased from Gibco (Grand Island, NY); fetal bovine serum (FBS) was obtained from Sijiqing (Hangzhou, China); mangiferin, horse serum, dimethyl sulfoxide (DMSO), and palmitic acid (PA) for cell experiments were obtained from Sigma-Aldrich (St. Louis, MO, USA); 3-(4,5-dimethylthiazol-2-yl)-2,5-diphenyl-2H-tetrazolium bromide (MTT) for cytotoxicity was purchased from MP Biomedicals (CA, USA); 2-deoxy-2-[(7-nitro-2,1,3-benzoxadiazol-4-yl)amino]-D-glucose (2-NBDG) for confocal microscopy experiments was obtained from Invitrogen Corporation (CA, USA); glucose transporter type 2 (GLUT2) and glucose transporter type 4 (GLUT4) were purchased from Abcam (Cambridge, UK); peroxisome proliferator-activated receptor *α* (PPAR*α*), PPAR*α* siRNA (h), antibody against fatty acid translocase (CD36), carnitine palmitoyltransferase 1 (CPT1), and *β*-actin were purchased from Santa Cruz Biotechnology (Santa Cruz, CA, USA); and AKT and P-AKT were obtained from Cell Signaling Technology (Boston, USA). The polyvinylidene difluoride (PVDF) membrane was obtained from Millipore Corp. (Billerica, MA).

### 2.2. Cell Culture and Treatment

The human hepatoma (HepG2) cell line and C2C12 myoblasts were obtained from the Chinese Academy of Sciences (Shanghai, China). The cells were maintained in DMEM containing 10% fetal bovine serum and 1% antibiotic/antimycotic at 37°C in an atmosphere containing 95% air and 5% CO_2_. To induce differentiation in the C2C12 myoblasts, after they were grown to confluence, the medium was switched to high glucose (~25 mM) DMEM containing 2% horse serum and changed every other day [14]. The differentiated C2C12 cells fused into multinuclear myotubes in 7–9 days. HepG2 cells and C2C12 myotubes were treated with 0.25 mM of PA and mangiferin (12.5, 25, and 50 *μ*M) or without PA and mangiferin (control) in serum-free medium with 1% FFA-free bovine serum albumin (BSA) for 24 h. The preparation method of PA was described in detail previously [15].

The study protocol has been approved by the Ethics Committee of the Harbin Medical University within which the work was undertaken, and it conforms to the provisions of the Declaration of Helsinki.

### 2.3. Cell Viability

The cell viability was measured with MTT and lactate dehydrogenase (LDH) assays (Roche Diagnostics GmbH Roche Applied Science, Mannheim, Germany) as previously described [16, 17].

### 2.4. Glucose Uptake Assay

The HepG2 cells and C2C12 myotubes were given 0.25 mM of PA and mangiferin (12.5, 25, and 50 *μ*M) simultaneously for 24 h. The fixed cells were washed three times with PBS, and subsequently, the cells were incubated in transport buffer at 37°C in the presence or absence of 100 nM insulin for 30 min before the addition of 100 *μ*L 2-NBDG (100 *μ*M) in the 24-well plate for 30 minutes at 37**°**C. The fluorescence was measured immediately at wave lengths of 488 nm for excitation and 550 nm for emission [18], or the data from 10,000 single cell events were collected using a flow cytometer (BD LSRFortessa) within 20 s for each measurement.

### 2.5. Glucose Content Measurement

The glucose content was measured by the Glucose Oxidase Assay Kit (APPLYGEN, Beijing, China). The level of glucose was calculated in *μ*mol/mg protein.

### 2.6. Determinations of Intracellular TG, Intracellular PA, and PA Uptake in the Medium

HepG2 cells and C2C12 myotubes were treated with 0.25 mM of PA and mangiferin (12.5, 25, and 50 *μ*M) or without PA and mangiferin (control) in serum-free medium with 1% FFA-free BSA for 24 h. The cellular lipid was extracted using a previously described method [19]. Cell protein was determined by the Bradford method [20]. The intracellular TG mass was quantified spectrophotometrically at 490 nm using a TG test kit (APPLYGEN, Beijing, China). The concentration of PA remaining in the medium after treatment was determined to assess the uptake of PA by HepG2 cells and C2C12 myotubes. PA was examined by GC-MS (TRACE GC/PolarisQ MS, Thermo Finnigan, Austin, USA) according to a previously described method by our laboratory [21].

### 2.7. Measurements of Intracellular AMP and ATP in HepG2 Cells and C2C12 Myotubes

After the treatment as described above, the method of cells pretreatment was described in detail by Budinger et al. [22]. The assay of measuring of intracellular AMP and ATP in HepG2 cells and C2C12 myotubes was described in detail previously [9].

### 2.8. Cell Transfection

The targeted siRNA was mixed with Lipofectamine 2000 (Invitrogen, Carlsbad, CA, USA) in buffer, as well as control siRNAs, which have no effect on the silencing of the gene expression, followed by incubation for 20 min at room temperature. We replaced the normal culture medium without antibiotics after transfection overnight. After 12 h, the HepG2 cells and C2C12 myotubes were treated with 0.25 mM of PA and 50 *μ*M of mangiferin for 24 h; the cells were then collected for measurement of the key protein expressions by western blotting.

HepG2 cells and C2C12 myotubes were transfected with pEX-RB-SOCS3 or pEX-RB-PTP1B recombinant plasmid (RiboBio, Guangzhou, China) using Lipofectamine 2000 for 48 h. Then the cells were treated with 0.25 mM of PA and 50 *μ*M of mangiferin for 24 h and AKT and P-AKT proteins were determined by western blot.

### 2.9. Fatty Acid *β*-Oxidation Rate Assessment

The HepG2 cells and C2C12 myotubes were given 0.25 mM of PA and mangiferin (12.5, 25, and 50 *μ*M) simultaneously for 24 h. The cell mitochondria were isolated integrally with the Cell Mitochondrial Isolation Kit (Beyotime, Shanghai, China). Then the mitochondria were used to assess the fatty acid *β*-oxidation rate according to the Fatty Acid *β*-Oxidation Kit from Genmed Scientifics Inc., USA. The *β*-oxidation rate was determined by measuring the reduction rate of palmitoyl carnitine oxidation-dependent ferricyanide.

### 2.10. Western Blotting Analysis

The proteins PPAR*α*, GLUT2, GLUT4, CD36, and CPT1 were determined by western blot analysis as previously described [17]. The primary antibodies were diluted in blocking buffer containing 1% BSA at 4°C overnight. Anti-goat or anti-rabbit alkaline phosphatase-conjugated antibody (Promega, Madison, WI, USA) was used as a secondary antibody.

### 2.11. Statistical Analysis

The Statistical Package of Social Sciences (SPSS) 18.0 software was used for all statistical analyses. Values were expressed as mean ± standard deviation. Statistical analyses were conducted by one-way ANOVA and pairwise comparisons using the Dunnett test, and *P* < 0.05 was considered to be statistically significant.

## 3. Results

### 3.1. Cell Viability

**Figure 1:**
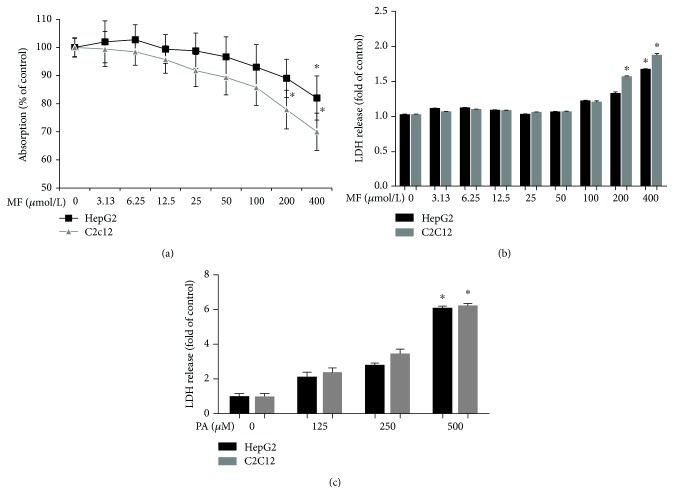
The cytotoxic effects of mangiferin and PA on HepG2 cells and C2C12 myotubes. The two cells lines were given 0, 3.13, 6.25, 12.5, 25, 50, 100, 200, and 400 *μ*M of mangiferin for 24 h. (a) MTT assay. (b) LDH release in culture media. (c) LDH release in culture media. The experiments were repeated 3 times. Data are presented as means ± SD (*n* = 3). ^∗^ *P* < 0.05.

HepG2 cells and C2C12 myotubes were treated with 0–400 *μ*M of mangiferin for 24 h to examine cell viability by MTT (Figure 1(a)) and LDH assays (Figure 1(b)). Cytotoxicity was observed at 400 *μ*M of mangiferin in HepG2 cells and 200 *μ*M in C2C12 myotubes. Therefore, mangiferin concentration was safe within 12.5–50 *μ*M in HepG2 cells and C2C12 myotubes in the present study. In addition, HepG2 cells and C2C12 myotubes were treated with 0–500 *μ*M of PA for 24 h to assess cytotoxicity by LDH (Figure 1(c)). The results suggest that the reasonable dose was 250 *μ*M to establish a lipotoxicity model mediated by high levels of FFA in HepG2 cells and C2C12 myotubes.

### 3.2. Mangiferin Improved Insulin Sensitivity in HepG2 Cells and C2C12 Myotubes

**Figure 2:**
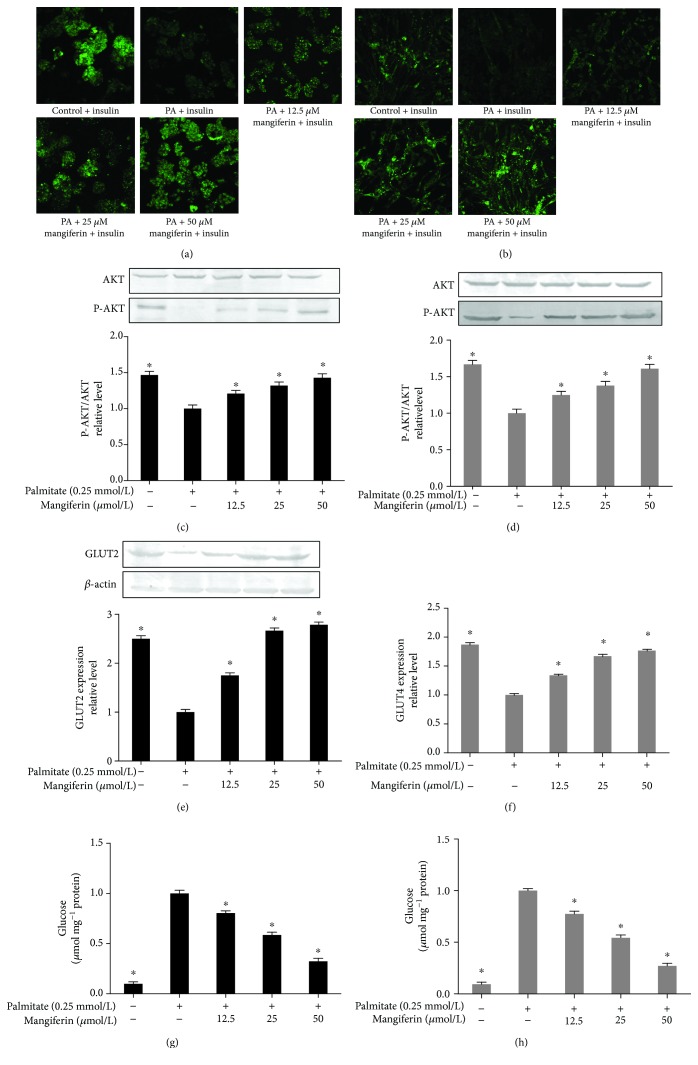
The glucose uptake, AKT, GLUT2 and GLUT4 expressions, and glucose content in HepG2 cells and C2C12 myotubes. HepG2 cells and C2C12 myotubes were treated with 0.25 mM of PA and 12.5, 25, and 50 *μ*M of mangiferin for 24 h. The cells were incubated in transport buffer in the presence of 100 nM insulin for 30 min before the addition of 100 *μ*L 2-NBDG for 30 min at 37°C. The glucose uptake (a–b), AKT (c–d), GLUT2 (e) and GLUT4 (f) protein expressions, and glucose content (g–h) in HepG2 cells and C2C12 myotubes. The experiments were repeated 3 times. Data are presented as means ± SD (*n* = 3). ^∗^ *P* < 0.05 compared with the PA group.

**Figure 3:**
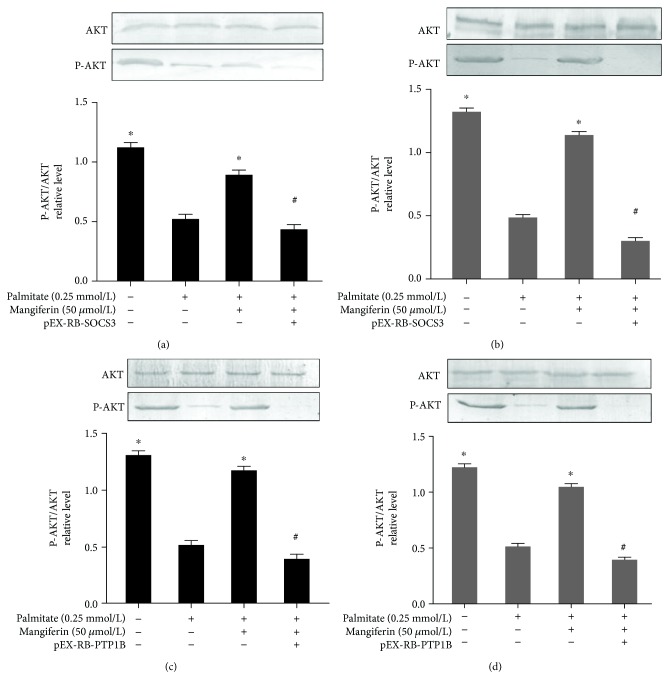
Effects of mangiferin on AKT expressions by regulating SOCS3 and PTP1B in HepG2 cells and C2C12 myotubes. The two cell lines were transfected with pEX-RB-SOCS3 or pEX-RB-PTP1B recombinant plasmid using Lipofectamine 2000 for 48 h. Then the cells were treated with 0.25 mM of PA and 50 *μ*M of mangiferin for 24 h. The expressions of AKT and P-AKT were determined by western blot method in HepG2 cells (a, c) and C2C12 myotubes (b, d). The experiments were repeated 3 times. Data are presented as means ± SD (*n* = 3). ^∗^ *P* < 0.05 compared with the PA group and ^#^ *P* < 0.05 compared with the mangiferin group.

We measured glucose uptake using 2-NBDG to determine whether mangiferin enhanced insulin sensitivity in IR cells. The glucose uptake was decreased markedly after treatment with 0.25 mM of PA, indicating that establishment of the IR model was due to the accumulation of PA. Furthermore, insulin infusion alone resulted in a marked increase in 2-DG uptake (Figure S1A-B), and mangiferin treatments significantly increased the insulin-stimulated glucose uptake in a dose-dependent manner (Figures 2(a) and 2(b)) and prevented PA-induced reduction of P-AKT, GLUT2, and GLUT4 expressions (Figures 2(c)–2(f)) and decreased glucose levels (Figures 2(g) and 2(h)) in HepG2 cells and C2C12 myotubes, indicating an enhanced P-AKT, GLUT2, and GLUT4 in response to insulin. Additionally, P-AKT expressions were obviously repressed by the inhibitor of insulin signaling SOCS3 or PTP1B in the presence of 0.25 mM of PA and 50 *μ*M of mangiferin in HepG2 cells and C2C12 myotubes (Figures 3(a)–3(d)). The results suggest that mangiferin regulates the inhibitor of the insulin pathway SOCS3 or PTP1B through P-AKT to improve IR.

### 3.3. Effects of Mangiferin on FFA and TG Levels in HepG2 Cells and C2C12 Myotubes

**Figure 4:**
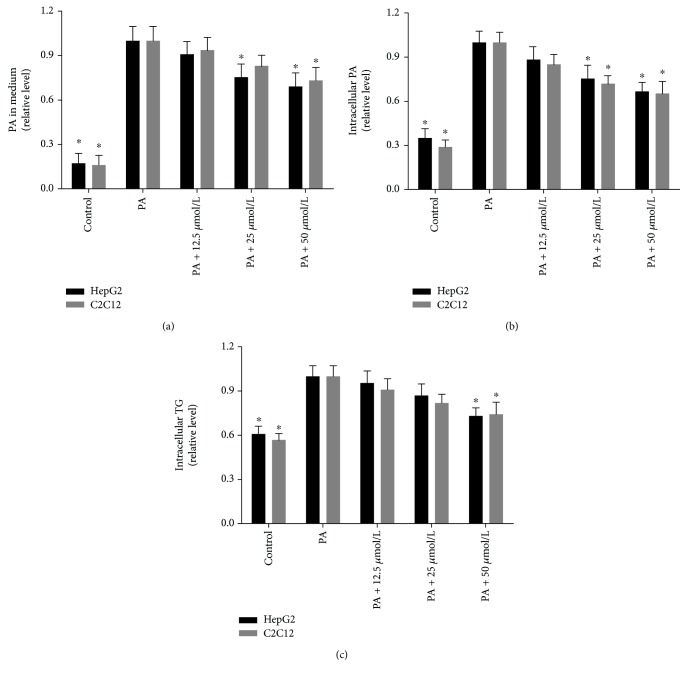
Effects of mangiferin on PA and TG in HepG2 cells and C2C12 myotubes. HepG2 cells and C2C12 myotubes were treated with 0.25 mM of PA and 12.5, 25, and 50 *μ*M of mangiferin for 24 h. The concentrations of PA in medium (a) and intracellular (b) PA were determined by GC-MS. The TG (c) mass was quantified by using a TG test kit. The experiments were repeated 3 times. Data are presented as means ± SD (*n* = 3). ^∗^ *P* < 0.05 compared with the PA stimulation group.

**Figure 5:**
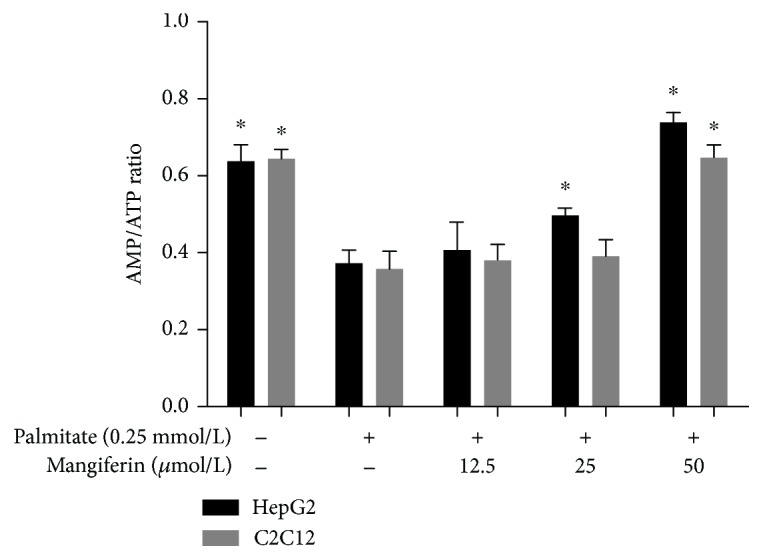
Effects of mangiferin on the ratio of AMP to ATP in HepG2 and C2C12 cells. The two cell lines were exposed to 0.25 mM of palmitate only or with 12.5, 25, and 50 *μ*M of mangiferin for 24 h. The experiments were repeated 3 times. Data are presented as means ± SD (*n* = 3). ^∗^ *P* < 0.05 compared with the PA group.

Mangiferin treatments significantly reduced the PA concentration of medium in a dose-dependent manner in HepG2 cells and C2C12 myotubes. PA uptake was calculated by the concentration of PA remaining in the medium after treatment; 25 and 50 *μ*M of mangiferin increased the PA uptake by 25% and 31%, respectively, in HepG2 cells; and 50 *μ*M of mangiferin increased the PA uptake by 27% in C2C12 myotubes (*P* < 0.05, Figure 4(a)). In addition, 25 and 50 *μ*M of mangiferin obviously reduced the intracellular PA content (*P* < 0.05, Figure 4(b)), and 50 *μ*M of mangiferin evidently decreased the intracellular TG content (*P* < 0.05, Figure 4(c)) in HepG2 cells and C2C12 myotubes. Moreover, mangiferin increased the ratio of AMP to ATP in a dose-dependent manner in HepG2 and C2C12 cells (*P* < 0.05, Figure 5), which suggests that oxidative phosphorylation increase partly due to energy-demanding process enhancement. These results reveal that mangiferin can ameliorate IR possibly by reducing the levels of FFA in medium and inhibiting TG accumulations of cells.

### 3.4. Mangiferin Regulated the Key Enzymes That Increase the FFA Uptake and Oxidation in HepG2 Cells and C2C12 Myotubes

**Figure 6:**
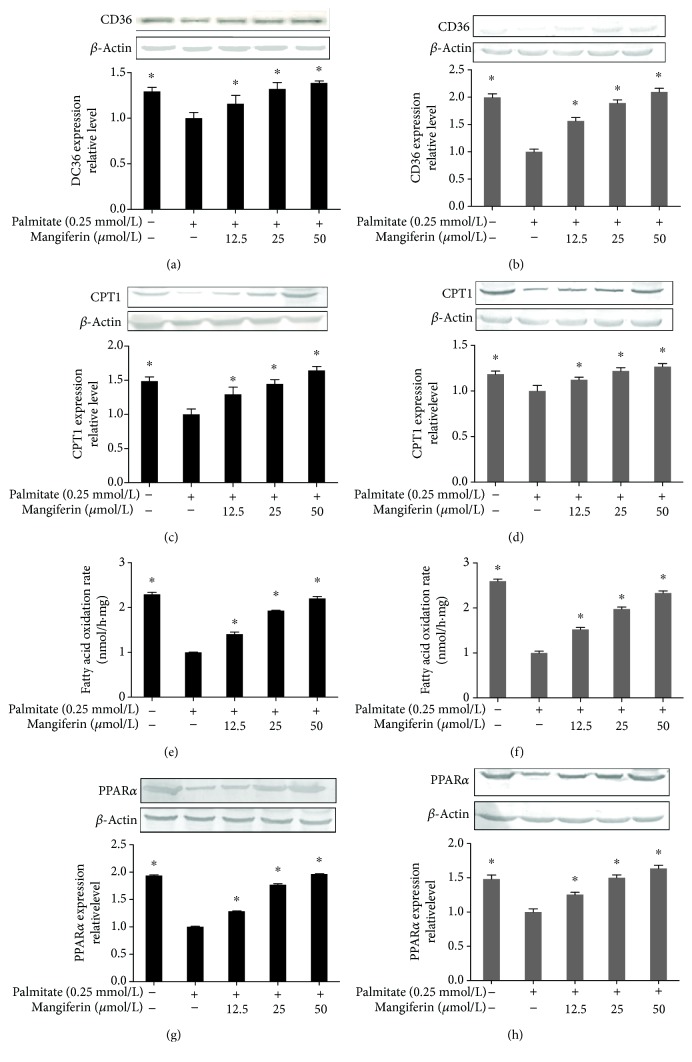
Effects of mangiferin on CD36, CPT1, FFA oxidation rate, and PPAR*α* in HepG2 cells and C2C12 myotubes. HepG2 cells and C2C12 myotubes were incubated with 0.25 mM of PA and 12.5, 25, and 50 *μ*M of mangiferin for 24 h. The CD36 and CPT1 expressions, FFA oxidation rate, and PPAR*α* expression in HepG2 cells (a, c, e, g) and C2C12 myotubes (b, d, f, h). The experiments were repeated 3 times. Data are presented as means ± SD (*n* = 3). ^∗^ *P* < 0.05 compared with the PA stimulation group.

We examined the key proteins of FFA metabolism including CD36 and CPT1 by mangiferin intervention to further verify the mechanism of mangiferin on FFA metabolism. The results indicated that CD36, which involves fatty acid uptake, and CPT1, which is the rate-limiting enzyme of fatty acid *β*-oxidation, were markedly restored by mangiferin in both HepG2 cells and C2C12 myotubes (*P* < 0.05, Figures 6(a)–6(d)). Meanwhile, mangiferin obviously increased the fatty acid *β*-oxidation rate in HepG2 cells and C2C12 myotubes (*P* < 0.05, Figures 6(e) and 6(f)). Furthermore, CD36 and CPT1 were increased at 50 *μ*M of mangiferin for 24 hours (*P* < 0.05, Figure S2A-D). In addition, PPAR*α* is a key mediator in meditating fatty acid uptake and oxidation and regulates CD36 and CPT1 protein expressions in the liver and skeletal muscle. The PPAR*α* expression was also potentiated by mangiferin in both HepG2 cells and C2C12 myotubes (*P* < 0.05, Figures 6(g) and 6(h)), which indicated that mangiferin improved the FFA catabolism by the PPAR*α* signaling pathway.

### 3.5. The PPAR*α* Pathway Contributed to Mangiferin Protection against IR in HepG2 Cells and C2C12 Myotubes

**Figure 7:**
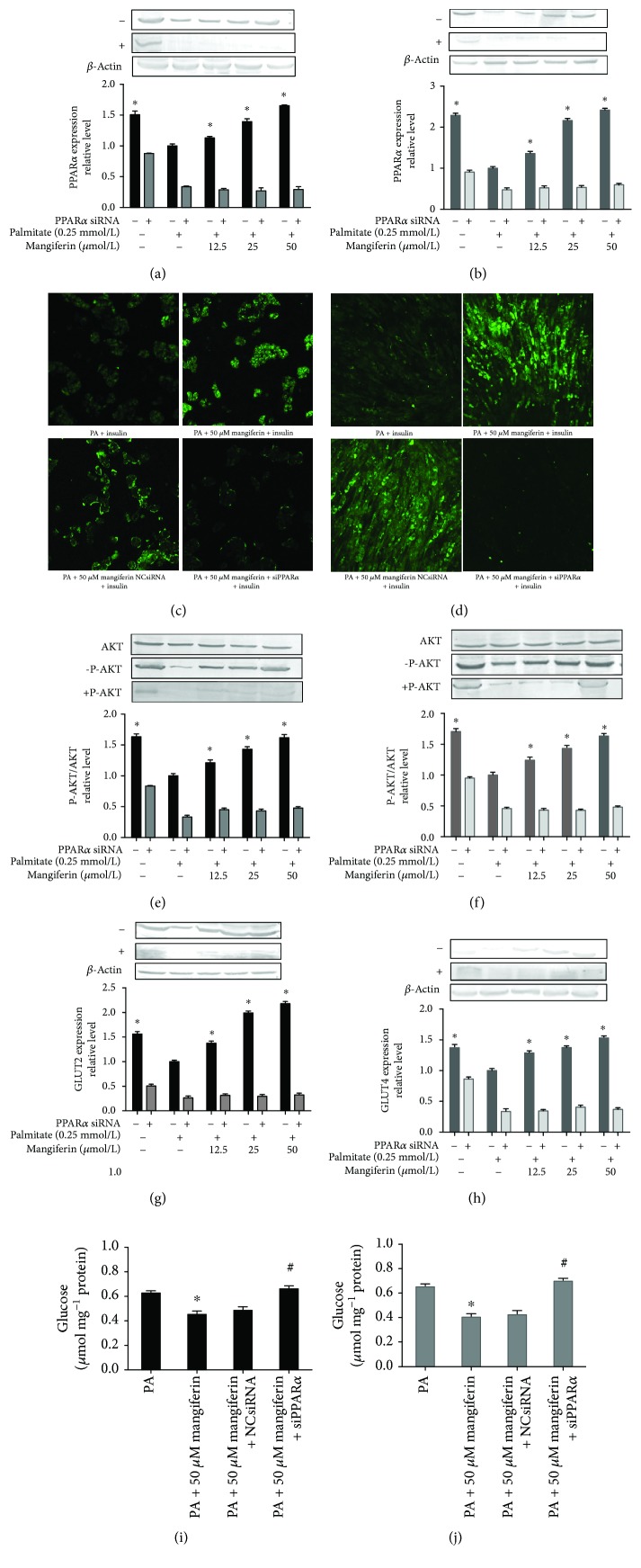
Effects of mangiferin on related indicators via siPPAR*α* in HepG2 cells and C2C12 myotubes. PPAR*α* expression, glucose uptake, AKT, GLUT2 and GLUT4 expressions, and glucose content in HepG2 cells (a, c, e, g, i) and C2C12 myotubes (b, d, f, h, j). The experiments were repeated 3 times. Data are presented as means ± SD (*n* = 3). ^∗^ *P* < 0.05 compared with the PA stimulation group.

**Figure 8:**
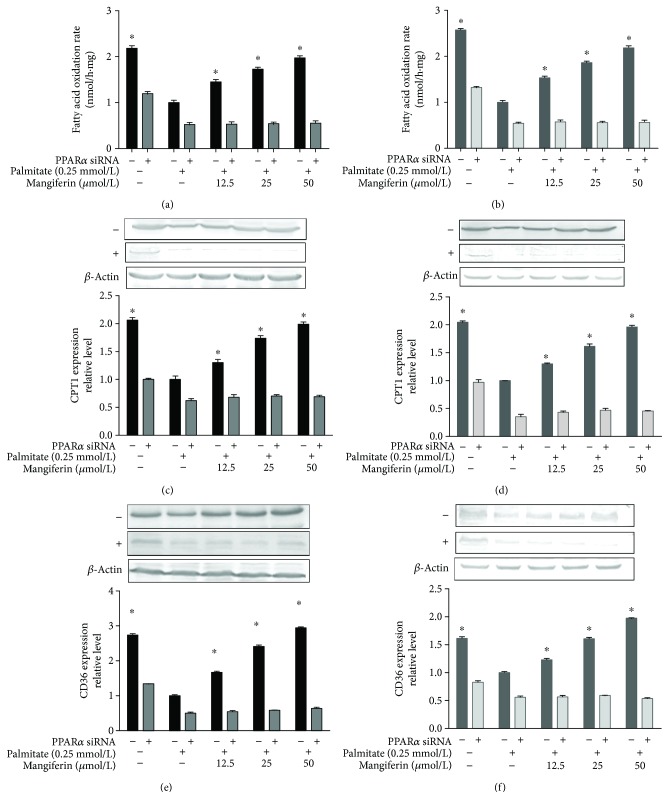
Effects of mangiferin on FFA oxidation rate, CPT1, and CD36 via siPPAR*α* in HepG2 cells and C2C12 myotubes. FFA oxidation rate, CPT1 expression, and CD36 expression in HepG2 cells (a, c, e) and C2C12 myotubes (b, d, f). The experiments were repeated 3 times. Data are presented as means ± SD (*n* = 3). ^∗^ *P* < 0.05 compared with the PA stimulation group.

To explicate the effect of PPAR*α* in modulating mangiferin reduction of high FFA-induced IR in HepG2 and C2C12 cells, the cells were treated with 0.25 mM of PA and 50 *μ*M of mangiferin, in the presence or absence of PPAR*α* siRNA. The PPAR*α* protein (Figures 6(a) and 6(b)), glucose uptake (Figures 7(c) and 7(d)), P-AKT (Figures 7(e) and 7(f)), and GLUT2 and GLUT4 expressions (Figures 7(g) and 7(h)) were also significantly attenuated, the glucose content was obviously increased (*P* < 0.05, Figures 7(i) and 7(j)), and the fatty acid *β*-oxidation rate was markedly decreased (Figures 8(a)–8(b)), as well as the expressions of PPAR*α* downstream proteins, including CPT1 and CD36, which were also suppressed noticeably in the presence of PPAR*α* siRNA in both HepG2 cells and C2C12 myotubes (*P* < 0.05, Figures 8(c)–8(f)). These results strongly suggest that mangiferin exerts its effect on ameliorating IR and promoting FFA uptake and oxidation via the PPAR*α* signaling pathway in both HepG2 cells and C2C12 myotubes.

## 4. Discussion

Mangiferin can exert an antidiabetic effect and improve IR in animal experiments with rats and mice [23]. However, there are few studies on the mechanism of mangiferin on mitigating IR *in vitro*. In addition, FFA is a major component of blood lipids and plays a critical role in regulating glucose and lipid metabolism. Elevated plasma FFA can lead to hyperlipidemia, IR, and T2DM. However, little is known about its mechanism of mangiferin on IR by FFA metabolism.

The liver and skeletal muscle are metabolically active tissues, mainly responsible for glucose metabolism and FFA oxidation. In this study, we successfully established the IR models of HepG2 cells and C2C12 myotubes induced by 0.25 mM of PA [24]. Our results showed that insulin infusion alone increased 2-DG uptake and mangiferin raised insulin-stimulated glucose uptake in a dose-dependent manner. AKT is a serine/threonine-specific protein kinase that plays a key role in multiple cellular processes such as glucose metabolism, cell proliferation, transcription, and cell migration [25]. GLUT2 is an integral membrane facilitative glucose transporter, and the substantial GLUT2 protein is located in the HepG2 cell membrane [26]. GLUT4 is a mammalian facilitative glucose transporter that is highly expressed in adipose tissue and striated muscle [27]. The results of the present study suggest that mangiferin prevents PA-induced reduction of glucose uptake, P-AKT, and GLUT2 and GLUT4 expressions and the enhancement of glucose levels in HepG2 cells and C2C12 myotubes. Moreover, mangiferin modulates SOCS3 and PTP1B that are negative regulators of the insulin pathway through phosphorylating AKT to increase insulin-induced cellular responses.

In addition, 15 mg/kg of mangiferin can mitigate IR in a rat model of fructose-induced metabolic syndrome, and Girón et al. report that *Salacia oblonga* extract including mangiferin increased glucose transporter 4-mediated glucose uptake in L6 rat myotubes [28]. These data collectively further showed that mangiferin ameliorated IR and promoted glucose transport to peripheral tissues, including the liver and skeletal muscle.

With glucose and fatty acids in competition as the energy supply, elevated fatty acid levels will depress glucose uptake in liver and skeletal muscle tissues [29]. Conversely, decreased fatty acid will promote glucose uptake, which increases glucose utilization and improves IR. In this study, 0.25 mM of PA increased significantly the intracellular TG content in liver and skeletal muscle cells, a condition that is observed often when plasma FFA is elevated in human and animal studies. Mangiferin significantly reduced the levels of intracellular FFA and TG in both HepG2 cells and C2C12 myotubes, which indicated that mangiferin promoted fatty acid uptake and oxidation instead of accumulation of intracellular TG. Therefore, we propose that mangiferin exerts the effect of ameliorating IR and enhancing glucose uptake by promoting FFA metabolism.

CD36 is a receptor for several ligands, including oxidized LDL and long-chain fatty acids, where it is the rate-limiting enzyme involved in high-affinity uptake of fatty acids. CPT1 is a mitochondrial enzyme responsible for the formation of acyl carnitines by catalyzing the transfer of the acyl group of a long-chain fatty acyl-CoA from coenzyme A to l-carnitine. Long-chain acyl-coenzyme A (LCACoA) must first be actively transferred from the cytoplasm into the mitochondria for FFA *β*-oxidation in the liver and skeletal muscle [30]. We have found that CD36 and CPT1 are involved in the increased FFA uptake and *β*-oxidation supplemented with mangiferin in PA-induced HepG2 cells and C2C12 myotubes in this study, a conclusion that is supported by the restoring of CD36 and CPT1 protein expressions and the fatty acid *β*-oxidation rate. Moreover, mangiferin increased the ratio of AMP to ATP in a dose-dependent manner in HepG2 and C2C12 cells, which suggests that the increase in oxidative phosphorylation occurs in muscle or liver cells partly due to an increase in energy-demanding processes. PPAR*α*, an upstream regulator of CD36 and CPT1, is a critical factor involved in glucose and fatty acid metabolism [31]. Results from this study verified that PPAR*α* protein expression was also markedly restored in PA-induced HepG2 cells and C2C12 myotubes. In addition, the effects of glucose uptake, glucose level, and fatty acid *β*-oxidation rate and the expressions of P-AKT, GLUT2, GLUT4, CD36, and CPT1 caused by mangiferin were significantly reversed by siRNA-mediated knockdown of PPAR*α* in both HepG2 cells and C2C12 myotubes. The results indicate that the mechanism of enhancement of FFA metabolism by mangiferin involves activation of the PPAR*α* signaling pathway. However, the means by which mangiferin causes an increase in PPAR*α* protein expression needs further study.

## 5. Conclusions

Our results have demonstrated that mangiferin has a beneficial effect on IR by improving insulin sensitivity and glucose uptake, and the possible mechanism is that mangiferin promotes FFA catabolism by regulating the key enzymes of FFA uptake and oxidation by the PPAR*α* signaling pathway.

## Data Availability

All data were uploaded as supporting information files accompanying the manuscript.

## Supplementary Materials

Supplementary Materials Figure S1: the glucose uptake in HepG2 cells and C2C12 myotubes. Figure S2: effects of mangiferin on CD36 and CPT1 in HepG2 cells and C2C12 myotubes.
