# Supplementary material for: Mangiferin Improved Palmitate-Induced-Insulin Resistance by Promoting Free Fatty Acid Metabolism in HepG2 and C2C12 Cells via PPARα: Mangiferin Improved Insulin Resistance
Source: J Diabetes Res. 2019 Jan 27;2019:2052675. doi: 10.1155/2019/2052675 (PMC6369470; doi:10.1155/2019/2052675)
Supplement: Supplementary Materials — Figure S1: the glucose uptake in HepG2 cells and C2C12 myotubes. Figure S2: effects of mangiferin on CD36 and CPT1 in HepG2 cells and C2C12 myotubes. [file 2052675.f1.docx]

**
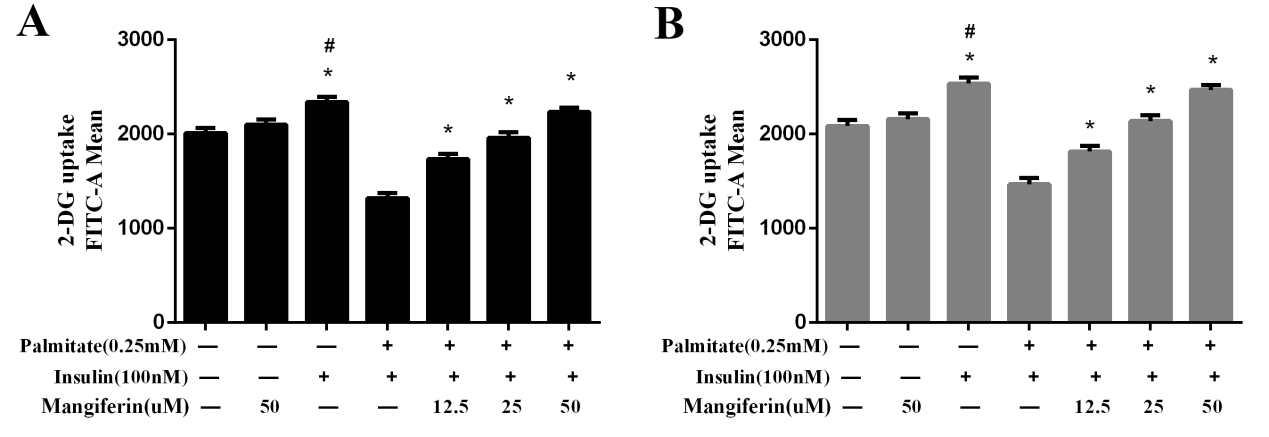
**

**Fig S1. The glucose uptake in HepG2 cells and C2C12 myotubes.** HepG2 cells and C2C12 myotubes were treated with 0.25 mM PA and 12.5, 25, 50 μM mangiferin for 24 h. The cells were incubated in transport buffer in the presence or absence of 100 nM insulin for 30min before the addition of 100 μL 2-NBDG for 30 min at 37°C. The glucose uptake was determined by flow cytometry in HepG2 cells (A) and C2C12 myotubes (B). The experiments were repeated 3 times. Data are presented as means ± SD (n=3). * *P* < 0.05 compared with PA group, #, *P* < 0.05 compared with control group.

**
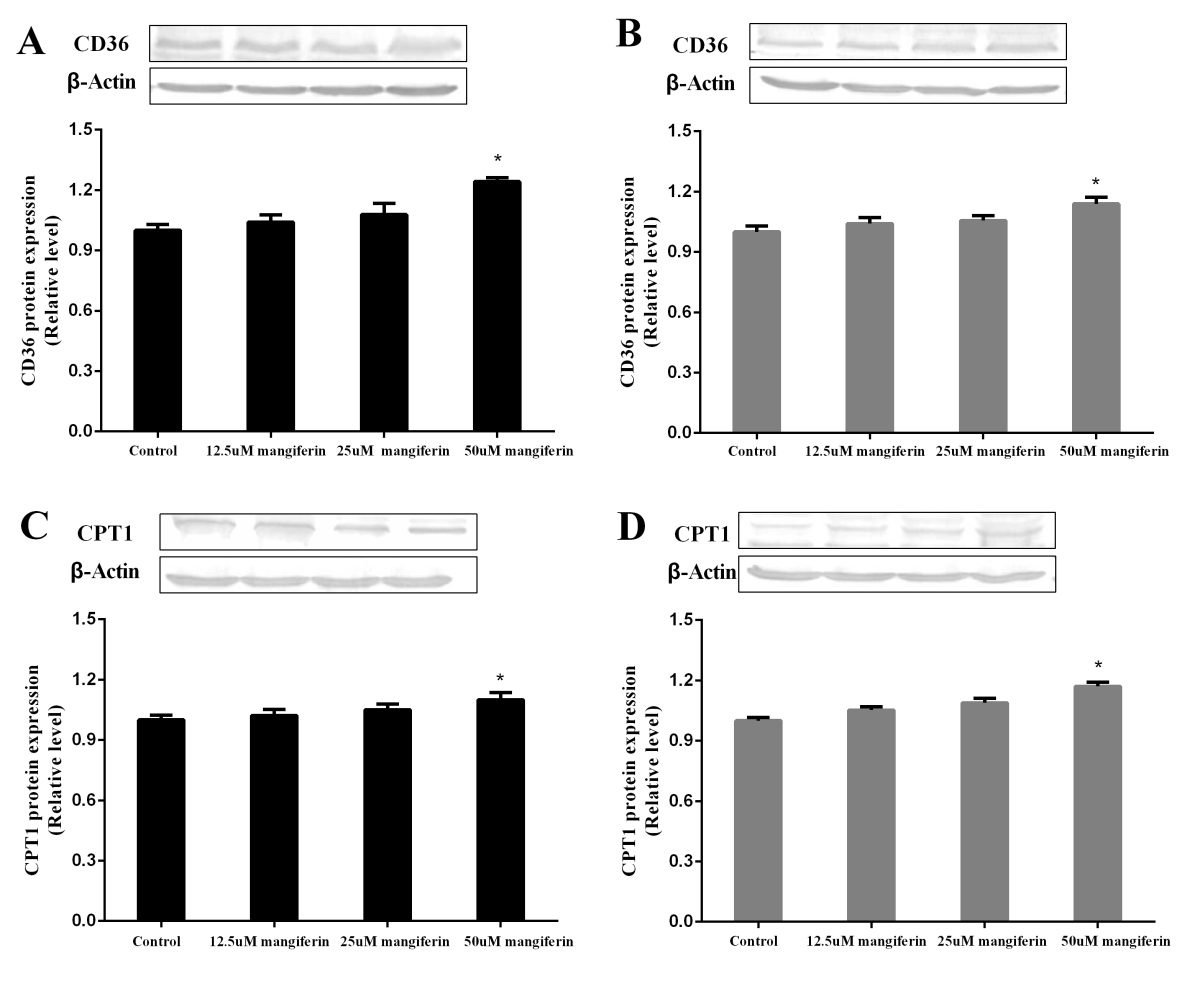
**

**Fig S2. Effects of mangiferin on CD36 and CPT1 in HepG2 cells and C2C12 myotubes.** The two cells lines were exposed to 12.5, 25 and 50 uM mangiferin simultaneously for 24h. The expressions of CD36 and CPT1 were determined by western blot method in HepG2 cells (A and C) and C2C12 myotubes (B and D). The experiments were repeated 3 times. Data are presented as means ± SD (n=3). * *P* < 0.05 compared with control group.
